# Blocking Host Factors IAP and DDX3 Activates HIV-1 Transcription and Increases Apoptosis Sensitivity of HIV-1 Infected Cells

**DOI:** 10.3390/pathogens15060575

**Published:** 2026-05-27

**Authors:** Jade Jansen, Shirley Man, Fenna Kootstra, Ad C. van Nuenen, Karel A. van Dort, Claudio Zamperini, Conraed Willem Houck, Neeltje A. Kootstra, Teunis B. H. Geijtenbeek

**Affiliations:** 1 Department of Experimental Immunology, Amsterdam UMC Location University of Amsterdam, 1105 AZ Amsterdam, The Netherlands; 2 Amsterdam Institute for Immunology and Infectious Diseases, 1105 AZ Amsterdam, The Netherlands; 3 First Health Pharmaceuticals B.V., 1098 XH Amsterdam, The Netherlands

**Keywords:** HIV-1 reservoir, latency reversal, SMAC mimetic, DDX3, non-canonical NF-κB, apoptosis

## Abstract

Antiretroviral therapy (ART) effectively suppresses HIV-1 replication but does not purge the latent HIV-1 reservoir. Strategies aimed at HIV-1 latency reversal and subsequent elimination of infected cells are being explored. Targeting the inhibitor of apoptosis proteins (IAP) and DEAD-box polypeptide 3 (DDX3) RNA helicase reduces the HIV-1 reservoir ex vivo. However, the mechanisms driving apoptosis of HIV-1 infected cells remain unclear. Here, we uncovered the mechanism regarding HIV-1 transcriptional activation and induction of apoptosis specific for HIV-1 infected cells using an acute in vitro infection model. Inhibition of IAP by second mitochondrial-derived activator of caspases mimetic (SMACm; AZD5582) resulted in activation of non-canonical NF-κB pathway (RelB/p52) that induced HIV-1 transcription, confirming previous reports, whereas inhibition of DDX3 sensitized HIV-1 infected cells for apoptosis (DDX3i; FH1321). Transcriptome analysis revealed that HIV-1 actively suppressed apoptosis-related genes in HIV-1 infected cells. SMACm treatment resulted in a broad induction of these genes irrespective of infection. Notably, DDX3 inhibition specifically restored the expression of the majority of HIV-1 suppressed genes, and when combined with SMACm, restored almost all HIV-1 downregulated genes, thereby rendering HIV-1 infected cells sensitive to apoptosis. Thus, our data strongly suggest that inhibition of host factors IAP and DDX3 not only induces activation of HIV-1 transcription but also restores HIV-1 suppressed apoptotic processes in infected cells.

## 1. Introduction

Antiretroviral therapy (ART) effectively suppresses HIV-1 replication and reduces the plasma viral load to undetectable levels. However, ART is not curative and discontinuation results in rapid viral rebound from the latent HIV-1 reservoir [1,2]. Therefore, HIV-1 cure strategies focusing on the elimination of the viral reservoir are needed.

The “shock and kill” method is one of the most investigated cure strategies and aims to purge the latent HIV-1 reservoir by reactivation of the latent provirus to restore viral transcription and protein production, followed by the targeted elimination of the infected cells [3,4]. Although latency reversal agents (LRAs) have been shown to enhance HIV-1 transcription, elimination of the reservoir remains difficult as LRAs have not been able to affect time to viral rebound or the size of the reservoir in vivo [5,6,7,8]. Therefore, it is likely that a combination of multiple agents is required to effectively reactivate and eliminate the reservoir [9].

Targeting host factors involved in HIV-1 replication processes might provide novel ways to reactivate HIV-1 or induce apoptosis of HIV-1 infected cells. The host factor RNA helicase DEAD-box polypeptide 3 (DDX3) is involved in the transport of HIV-1 mRNA from the nucleus [10] and has been shown to reactivate HIV-1 in latency models and induce selective cell death in HIV-1 infected cells [11], strongly suggesting DDX3i might act as an effective LRA. The inhibitor of apoptosis proteins (IAPs) suppress apoptosis [12]. Recently, we have shown that blocking DDX3 and IAPs with a DDX3 inhibitor (DDX3i) and second mitochondrial-derived activator of caspases mimetic (SMACm), respectively, leads to reactivation of latent HIV-1 provirus and significantly decreases the inducible latent HIV-1 reservoir in PBMCs from people living with HIV-1 (PWH) ex vivo [13]. Although the combination of SMACm and DDX3i has proven effective for inducing specific cell death in latent HIV-1-infected cells, the underlying mechanisms remain unclear.

Here, we identify the molecular mechanisms underlying HIV-1 transcriptional activation by SMACm and DDX3i and investigate their effect using a targeted qPCR array assay on apoptotic and cell survival processes in an acute in vitro infection model. Our data show SMACm induced HIV-1 transcription alone and in combination with DDX3i. Moreover, we demonstrated that SMACm and DDX3i increase the expression of apoptosis-related genes whose expression is specifically suppressed in HIV-1 infected cells, thereby sensitizing HIV-1 infected cells to apoptosis. Our findings shed light on the molecular requirements for HIV-1 cure strategies and suggest that combining HIV-1 transcriptional reactivation with the reversal of virus-induced anti-apoptotic pathways will enhance therapeutic efficacy.

## 2. Materials and Methods

### 2.1. Cell Cultures

J-lat cells (clone A1) and SUPT1-CCR5 cells were sourced from the NIH HIV Reagent Program, Division of AIDS, NIAID, NIH [14]. The J-lat A1 cell line contains an integrated HIV-1 LTR that drives expression of Tat and GFP [15,16]. The HeLa-derived HIV-1 reporter cell line TZM-BL, which carries β-galactosidase and firefly luciferase genes under the control of the HIV-1 long terminal repeat, was also obtained from the same program [17]. Cell lines were maintained in Iscove’s Modified Dulbecco’s Medium (IMDM; Thermo Fisher Scientific, Gibco, Waltham, MA, USA) supplemented with 10% fetal calf serum (FCS; HyClone, Cytiva, Marlborough, MA, USA) and antibiotics (100 U/mL penicillin and 100 µg/mL streptomycin) (Invitrogen, Carlsbad, CA, USA), and incubated at 37 °C in a humidified atmosphere with 10% CO_2_. HEK293T (ATCC Cat# CRL-3216) were cultured in Dulbecco’s Modified Eagle Medium (DMEM; Thermo Fisher Scientific, Gibco) containing 10% heat-inactivated FCS and the same antibiotics, under identical incubation conditions.

### 2.2. Compounds

The SMACm AZD5582 (Tebu-Bio, Le Perray en Yvelines, France) was prepared as a 10 mM stock solution in DMSO and stored at −80 °C. For experiments, it was diluted in culture medium to final concentrations ranging from 0.5 to 2 µM. The DDX3 inhibitor FH1321, provided by First Health Pharmaceuticals (Amsterdam, The Netherlands), was dissolved in DMSO at 20 mM and used at a final concentration of 50 µM after dilution in culture medium. TNF-α (PeproTech, Londen, UK) was reconstituted in medium at 1 mg/mL and stored at −20 °C. Prior to use, it was diluted to 50 ng/mL in culture medium. The NIK inhibitor Amgen16 (Merck, Darmstadt, Germany) was dissolved in DMSO at a 10 mM concentration and stored at 4 °C. Amgen16 was diluted in culture medium to final concentrations of 0.2–10 µM. Venetoclax (LKT Laboratories, St. Paul, MN, USA) was dissolved in DMSO at a 10 mM concentration and diluted in culture medium to a final concentration of 10 µM.

### 2.3. Flowcytometry

Expression of mKO2 and GFP in SUPT1-CCR5 cells infected with HIV-1 (HIV-1 GKO/VSV-G), as well as GFP expression in J-lat A1 cells, was assessed by flowcytometry. Cells were washed with phosphate-buffered saline (PBS, Thermo Fisher Scientific, Gibco) and fixed using FluoroFix™ Buffer (Biolegend, San Diego, CA, USA) following the manufacturer’s instructions. Caspase-3/7 activity was determined in SUPT1-CCR5 cells infected with HIV-1 NL4-3BaL using the FAM FLICA™ Caspase-3/7 Kit (Bio-Rad, Hercules, CA, USA) according to the manufacturer’s instructions. For intracellular p24 staining, the cells were fixed and permeabilized using Cytofix/Cytoperm™ Fixation/Permeabilization Kit (BD Biosciences, Franklin Lakes, NJ, USA) according to the manufacturer’s protocol and stained using p24-FITC (anti-HIV-1, 1:200, KC57, Beckman Coulter, Brea, CA, USA).

Fluorescence was measured with the BD FACSymphony™ A1 (BD Biosciences, Franklin Lakes, NJ, USA). Flowcytometry data was analyzed using FlowJo version 10 (BD Biosciences).

### 2.4. Virus Production and Infection Assays

The HIV-1 NL4-3BaL plasmid was obtained from the NIH AIDS Reagent Program [18], while the HIVGKO construct was kindly provided by Eric Verdin (Addgene plasmid # 112234) [19]. HIV-1 NL4-3BaL and HIV-1 GKO/VSV-G viral stocks were generated by transfecting HEK293T cells with plasmids encoding the respective proviral DNA using a calcium phosphate–based method.

For transfection in a 6-well plate, plasmid DNA was diluted in 0.042 M HEPES containing 0.15 M CaCl_2_, followed by the addition of an equal volume of 2× HBS (HEPES buffered saline) with gentle mixing. After a 15 min incubation, the mixture was added dropwise to HEK293T cells, which were then incubated overnight at 37 °C in a humidified atmosphere with 3% CO_2_. The next day, the culture medium was refreshed, and the transfected cells were further maintained at 37 °C with 10% CO_2_. Viral supernatants were collected at 2 and 3 days post-transfection, filtered through a 0.22 µm membrane, and stored in aliquots at −80 °C. Viral titers were determined using TZM-BL cells by calculating the TCID_50_.

SUPT1-CCR5 or TZM-BL cells were infected with either HIV-1 GKO/VSV-G or HIV-1 NL4-3BaL. After 48 h, the virus was removed from the culture, and cells were cultured in the presence of the compounds (where indicated) for at least 24 h before being prepared for further analysis: flowcytometry analysis (after 24 h) or luciferase expression measuring luminescence (after 48 h).

HIV-1 is classified as a Risk Group 3 pathogen. All experiments involving this virus have been performed in a certified biosafety cabinet within a Biosafety Level 3 (BSL-3) laboratory. Personnel involved in this work were appropriately trained and complied with all applicable local biosafety regulations in consultation with the local biosafety officer.

### 2.5. qPCR Analysis

For the qPCR, SUPT1-CCR5 cells were either infected with HIV-1 NL4-3BaL (MOI 0.3) or left uninfected for two days. Afterward, the virus was washed away, and the cells were incubated with the different compounds for six hours, whereafter the cells were lysed in RLT buffer and stored at −20 °C for later use in qPCR analysis. RNA was isolated using the RNeasy Plus Mini Kit (QIAGEN, Hilden, Germany) according to the manufacturer’s instructions. cDNA was produced using the M-MLV Reverse Transcriptase kit (Promega, Madison, WI, USA) and random hexamers (Promega). PCR arrays were performed for the different treatment conditions with or without HIV-1 infection, with the 384-well plates Apoptosis and Survival Tier 1–4 from Bio-Rad (Hercules, CA, USA), where 384 primer pairs are lyophilized on the plate. cDNA was diluted to a final concentration of 1 ng/µL, and qPCR was performed using 2× GoTaq^®^ qPCR Master Mix (Promega, Madison, WI, USA) on the LightCycler 480 System (Roche, Basel, Switzerland). The PCR was performed under the following cycling conditions: denaturation: 95 °C for 2 min; amplification: 40 cycles of 95 °C for 5 s and 60 °C for 30 s, followed by a melting curve stage. LightCycler 480 1.5.1.62 SP3 software (Roche, Basel, Switzerland) was used for analysis of relative copy numbers and melting curves. TATA-binding protein (TBP), Glyceraldehyde 3-phosphate dehydrogenase (*GAPDH*) and hypoxanthine phosphoribosyltransferase 1 (HPRT1) were included as reference genes to correct for cDNA input.

RT qPCRs using specific primer pairs to detect gene expression of individual genes (Supplementary Table S8) were performed using 2× GoTaq^®^ qPCR Master Mix (Promega, Madison, WI, USA) on the QuantStudio 5 device (Thermo Fisher Scientific, Waltham, MA, USA). The following cycling conditions were used: denaturation: 95 °C for 20 s; amplification: 40 cycles of 95 °C for 1 s and 60 °C for 20 s, followed by a dissociation step to 95 °C with a ramp rate of 0.075 °C/sec in a melting curve stage. The QuantStudio Design & Analysis 2 software (Thermo Fisher Scientific, Waltham, MA, USA) was used for analysis. *GAPDH* was included as a reference gene.

MS/SS/US HIV-1 RNA PCRs were carried out similarly, but cells were lysed after 8 h for MS RNA measurements, after 24 h for SS RNA measurements, and after 48 h for US RNA measurements. Specific primer pairs were used (Supplementary Table S8) and the following cycling conditions: denaturation: 95 °C for 10 min; amplification: 50 cycles of 95 °C for 10 s, 58 °C for 20 s, and 72 °C for 30 s, followed by a melting curve stage. *GAPDH* and β-actin (*ACTB*) were included as reference genes.

### 2.6. NF-κB Transcription Factor Binding Assay

Nuclear extracts were obtained from SUPT1-CCR5 cells after 24 h treatment with various compounds using a nuclear extraction kit (Active Motif, Carlsbad, CA, USA) according to the manufacturer’s instructions. The nuclear extract proteins were measured using the Pierce BCA protein assay kits (Thermo Fisher Scientific, Waltham, MA, USA) and subsequently used to measure NF-κB p65, p50, p52 and RelB transcription factor binding with the TransAM NF-κB family kit (Active Motif), following the manufacturer’s protocol. Absorbance of the samples was measured at 450 nm, and the background at 620 nm was subtracted from the measurements. Transcription factor binding activity was calculated relative to the internal positive control provided with the kit.

### 2.7. Cytochrome C Analysis

SUPT1-CCR5 cells were infected and treated as described above. After 24 h, cell lysates were prepared for Cytochrome C analyses using the Human Cytochrome c ELISA Kit (Thermo Fisher Scientific, Waltham, MA, USA) according to the manufacturer’s instructions. In this assay, cells are lysed under conditions that maintain the mitochondria intact, and therefore, cytosolic cytochrome c serves as a marker for mitochondrial outer membrane permeabilization.

### 2.8. Data Analysis and Statistics

Data was analyzed with RStudio 4.3.2, Morpheus (Broad Institute, Cambridge, MA, USA), and Graphpad Prism 10.2.3 (Graphpad Software Inc., San Diego, CA, USA). The cycle threshold (Ct) values from the PCR array data were first filtered, where genes with blank values were excluded from the data. Subsequently, 2^−ΔΔCt^ values, relative to both the average of the three reference genes and the uninfected untreated condition, were calculated. Hierarchical clustering was performed based on one minus Pearson correlation. Subsequently, differentially expressed genes were determined, where genes with 2^−ΔΔCt^ values <0.5 or >2 were considered. Pathway overrepresentation was performed using Reactome Pathway Analysis [20,21].

## 3. Results

### 3.1. SMACm Activates HIV-1 LTR Transcription

**Figure 1:**
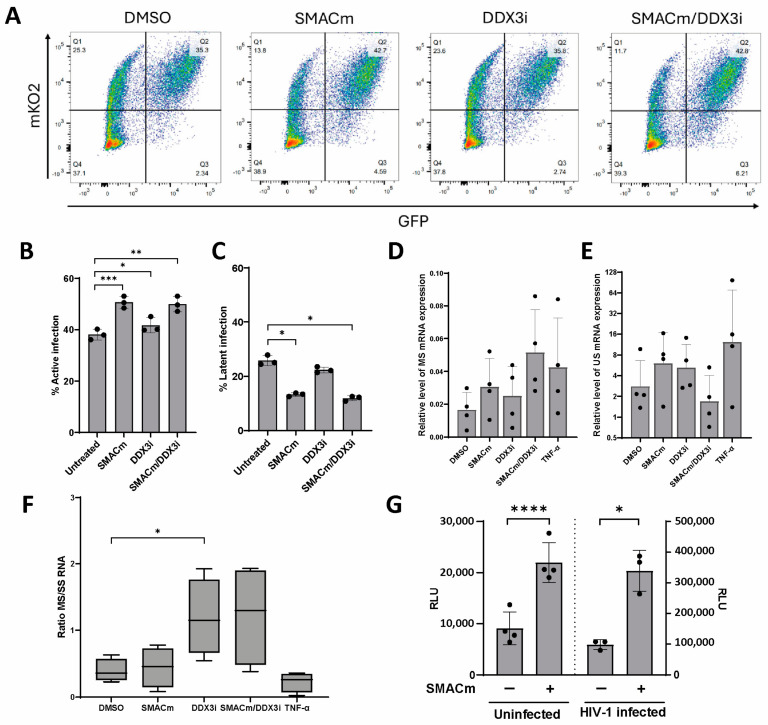
SMACm activates the HIV-1 LTR transcription. SUPT1-CCR5 cells were infected with the dual-reporter virus HIV-1 GKO-VSV-G and treated for two days with either SMACm (AZD5582; 1 µM), DDX3i (FH1321; 50 µM), or a combination of both. (**A**) Flowcytometry gating strategy for the analysis of mKO2 expression, representative of all HIV-1 infected cells, and GFP expression, representative of transcriptionally active HIV-1. The proportion of infected cells harboring transcriptionally active proviruses (**B**) or inactive proviruses (**C**) was determined by flowcytometry (N = 3). (**D**) SUPT1-CCR5 cells infected with HIV-1 NL4-3BaL were treated with SMACm (AZD5582; 1 µM), DDX3i (FH1321; 50 µM), or both; MS RNA was quantified at 8 h and US RNA (**E**) at 48 h post-treatment by qPCR (N = 4). (**F**) Ratio of MS to SS RNA transcripts after compound treatment (N = 4). (**G**) TZM-BL cells were either left uninfected or infected with HIV-1 NL4-3BaL (MOI 0.01) and then treated with or without SMACm (AZD5582; 1 µM) for two days. HIV-1 LTR activation was assessed by luminescence, reported in relative light units (RLU) (N = 4, uninfected, N = 3, HIV-1 infected). Average and standard deviation of independent experiments are shown. Symbols indicate the result of the independent experiment. Comparisons to untreated control were made using a paired *t*-test. Only significant events are displayed, * *p* < 0.05, ** *p* < 0.01, *** *p* < 0.001, **** *p* < 0.0001.

We analyzed whether SMACm (AZD5582) and DDX3i (FH1321) regulate HIV-1 transcription in an acute in vitro infection model. SUPT1-CCR5 cells were infected with the dual reporter virus HIV-1 GKO/VSV-G, and after 24 h, infected cells were treated with SMACm and DDX3i alone or in combination. HIV-1 transcription was determined by flowcytometry after 48 h: HIV-1 infected cells were detected by EF1α-driven mKusabira-Orange2 (mKO2) expression, and active HIV-1 transcription was assessed through HIV-1 LTR-driven GFP expression. In the untreated condition, 63.8% (±0.9) of cells were infected, with 38.1% (±2.1) harboring transcriptionally active and 25.7% (±1.8) transcriptionally inactive HIV-1 (Figure 1A–C). We also observed mKO2-negative/low GFP-positive cells, which most likely represent cells that went through a transcriptionally active state in which high levels of GFP have been produced. These mKO2-negative/low GFP-positive cells were relatively small, and this population was not analyzed separately. Moreover, due to differences in the half-life of fluorescent proteins, relatively high GFP expression is observed in cells with very low expression of mKO2 [22,23]. DDX3i treatment alone resulted in a slight increase in the proportion of cells with a transcriptionally active provirus (41.7% ± 3), but no change in the proportion of cells with transcriptionally inactive HIV-1 (22.3% ± 1.1) was observed. Treatment with SMACm alone or in combination with DDX3i strongly increased the proportion of cells with transcriptionally active HIV-1 (50.6% ± 2.3 and 50% ± 3, respectively) while reducing those with a transcriptionally inactive HIV-1 (13.2% ± 0.7 and 11.8% ± 0.9, respectively) (Figure 1A–C). These data strongly suggest that SMACm alone or in combination with DDX3i induces HIV-1 transcription as the proportion of cells with transcriptionally active HIV-1 increased significantly compared to that of untreated cells. To further confirm activation of transcription by SMACm, we measured HIV-1 transcription at the RNA level by quantifying both multiple spliced (MS) (Figure 1D) and unspliced (US) (Figure 1E) HIV-1 transcripts in the acute in vitro infection model. SMACm treatment increased both MS and US RNA levels, confirming transcriptional activation (Figure 1D,E). To assess whether DDX3i affects HIV-1 RNA processing and nuclear export, we analyzed the ratio of single spliced (SS, Rev-dependent) to MS (Rev-independent) transcripts following treatment. DDX3i treatment resulted in a shift towards increased MS relative to SS transcripts, indicating impaired nuclear export of Rev-dependent transcripts (Figure 1F).

We next investigated whether SMACm-induced activation of HIV-1 transcription depends on the HIV-1 transcriptional activator Tat, using the TZM-BL reporter cell line, in which luciferase expression is regulated by the HIV-1 LTR [17]. SMACm treatment in the absence of HIV-1 significantly increased LTR-driven luciferase activity in TZM-BL (Figure 1G; Supplementary Figure S1). Notably, although direct infection of TZM-Bl cells with HIV-1 NL4-3BaL increased luciferase expression tenfold compared to uninfected TZM-BL cells, SMACm treatment further increased luciferase expression (Figure 1G). These data suggest that SMACm initiates HIV-1 LTR-mediated transcription in acute infection, which is further enhanced by HIV-1 Tat. DDX3i treatment did not activate LTR-driven transcription in TZM-BL (Supplementary Figure S1).

### 3.2. SMACm Activates the HIV-1 LTR Primarily Through RelB by Preventing NIK Degradation

**Figure 2:**
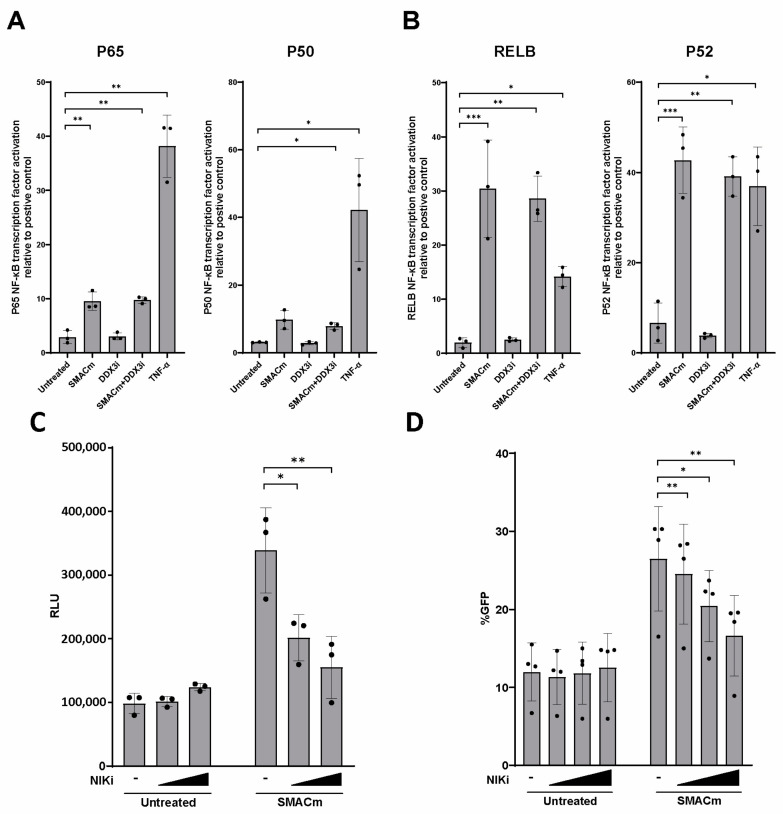
SMACm activates the HIV-1 LTR primarily through RelB by preventing NIK degradation. SUPT1-CCR5 were treated with SMACm (AZD5582; 1 µM), DDX3i (FH1321; 50 µM), both, or 50 ng/mL TNF-α for 24 h, and NF-κB transcription factor binding was determined by ELISA. Canonical NF-κB (p65/p50) transcription factor binding (**A**) and non-canonical NF-κB (RelB/p52) transcription factor binding (**B**) were measured (N = 3). Data has been normalized relative to the positive control supplied in the assay. TNF-α is used as a positive control for the induction of canonical NF-κB (p65/p50) and non-canonical NF-κB (RelB/p52) binding. (**C**) TZM-BL cells were infected with HIV-1 NL4-3BaL (MOI 0.01) and treated with SMACm (AZD5582; 1 µM), and NIKi (Amgen16; 5 and 10 µM), alone or in combination for two days. HIV-1 LTR activation was assessed by luminescence expressed in relative light units (RLU) (N = 3). (**D**) J-Lat A1 cells were treated with SMACm (AZD5582; 1 µM) with or without NIKi (Amgen16; 0.2–5 µM) for 2 days, and reversal of viral latency was analyzed by GFP expression (N = 4). Average and standard deviation of independent experiments are shown. Symbols indicate the result of the independent experiment. Comparisons to (untreated) controls were performed using a paired *t*-test. Significance is displayed, * *p* < 0.05, ** *p* < 0.01, *** *p* < 0.001.

Next, we investigated how SMACm induces HIV-1 transcription by investigating NF-κB activation as the important driver for HIV-1 transcription [24]. SUPT1-CCR5 cells were treated with SMACm alone or in combination with DDX3i, and nuclear translocation and activation of canonical p65 and p50, and non-canonical RelB and p52 subunits were determined. Cells were treated with compounds, and nuclear extracts were isolated, and the presence of activated NF-κB dimers was measured by TransAM NF-κB family assay. Activation in the assay was calculated relative to the internal control. TNF-α was taken along as the positive control for the activation of the different NF-κB subunits (Figure 2A,B). SMACm induced activation and nuclear translocation of the p65 and p50 subunits, albeit to a lesser extent than TNF-α (Figure 2A). Notably, SMACm induced nuclear translocation and activation of the non-canonical NF-κB subunits RelB and p52 more efficiently than TNF-α (Figure 2B). DDX3i did not induce p65/p50 or Rel/p52, nor did it affect SMACm-induced NF-κB activation (Figure 2A,B). These data suggest that SMACm induces activation of both canonical and non-canonical activation of NF-κB pathways, resulting in p65/p50 and even higher levels of RelB/p52 active dimers, which confirms previous reports [25,26,27].

In order to investigate whether SMACm-induced HIV-1 transcription is driven by non-canonical activation of NF-κB, we treated cells with a NIK inhibitor (NIKi), as SMACm mediates IAP degradation, which leads to NIK accumulation and subsequent activation of non-canonical activation of NF-κB [28,29]. NIK specifically targets the p100 to p52 processing. TZM-BL cells were infected with HIV-1 NL4-3BaL and subsequently treated with SMACm in the presence or absence of NIKi. SMACm induced HIV-1 LTR-driven luciferase expression, which was strongly decreased by inhibition of NIK (Figure 2C). As we have previously observed, SMACm reverses HIV-1 latency in different cell models [13]. We investigated whether non-canonical activation of NF-κB was involved in latency reversal using the HIV-1 latency model J-lat A1 cell line, which contains the GFP gene under the control of the HIV-1 LTR. SMACm induced GFP expression in J-lat A1, and the induction was decreased by NIKi in a dose-dependent manner (Figure 2D). Overall, these findings indicate that SMACm-induced HIV-1 transcription during infection and latency reversal is largely regulated through activation of the non-canonical activation of the NF-κB pathway.

### 3.3. SMACm and DDX3i Induce Distinct Expression Profiles of Apoptosis and Survival Genes in HIV-1 Infected and Uninfected SUPT1-CCR5 Cells

We have previously shown that the combination of SMACm and DDX3i induces cell death specifically of HIV-1 infected but not uninfected PBMCs from PWH [13]. To explore the underlying mechanism, we analyzed the effect of HIV-1 infection and the different compounds on the expression of genes involved in the survival/apoptosis pathway. Using the SUPT1-CCR5 acute in vitro infection model, we first confirmed by measuring the Caspase-3/7 activity that the compounds did not induce excessive apoptosis in either infected or uninfected cells, indicating limited toxicity (Supplementary Figure S2; [13]).

**Figure 3:**
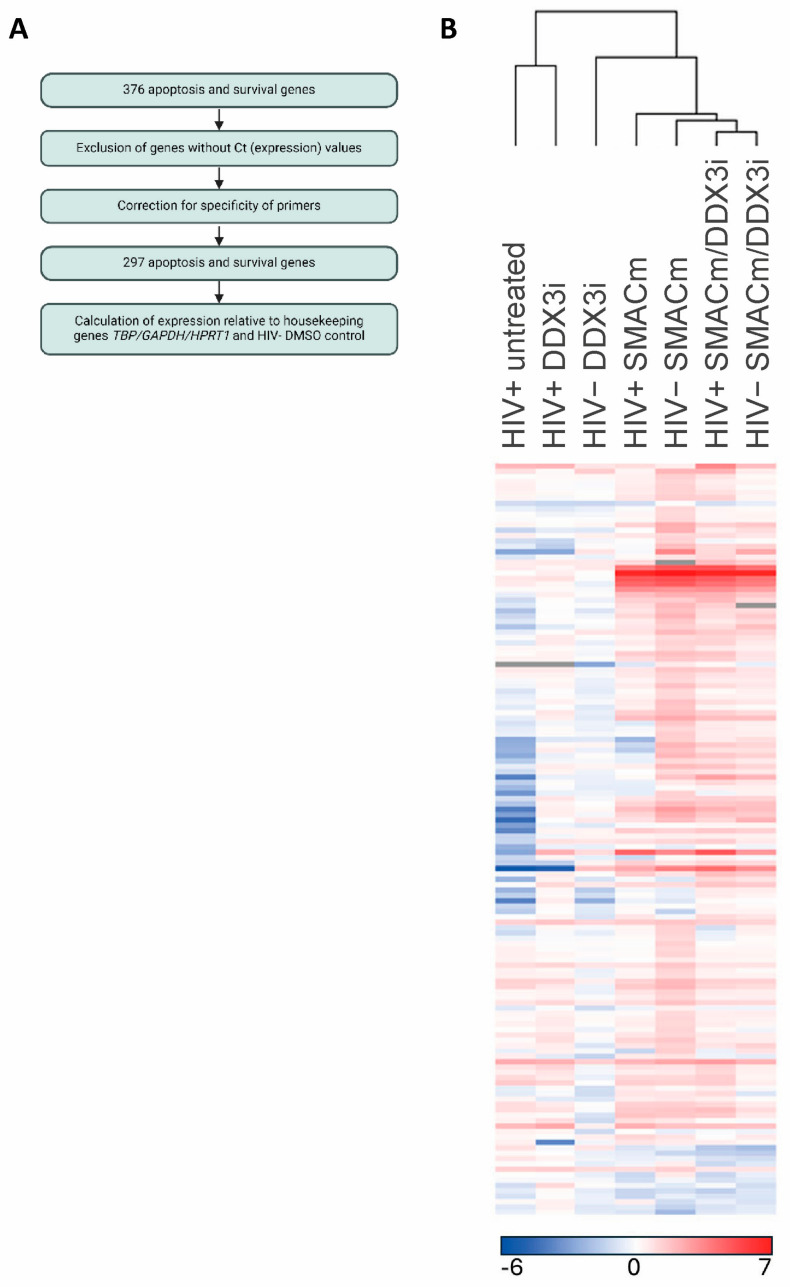
SMACm and DDX3i induce distinct expression profiles of apoptosis and survival genes in HIV-1 infected and uninfected SUPT1-CCR5 cells. (**A**) Apoptosis and survival qPCR arrays of SUPT1-CCR5 cells (infected with HIV-1 NL4-3 BaL or uninfected) treated with SMACm (AZD5582; 1 µM), DDX3i (FH1321; 50 µM), or both for 6 h or left untreated. In total, 376 genes were measured by qPCR. After quality control, 297 genes were included in the analysis. (**B**) Hierarchical clustered heatmap displaying log2-transformed fold change in gene expression as compared to uninfected and untreated SUPT1-CCR5 cells, with each column representing a condition and each row representing a gene. Values range from −6 to 7 as reflected by the color bar, with downregulated gene expression in blue and upregulated gene expression in red. The value of zero (white) indicates unchanged gene expression compared to uninfected and untreated SUPT1-CCR5 cells. Gray indicates gene expression levels below the level of detection.

Next, SUPT1-CCR5 cells infected for 48 h with full-length replication-competent HIV-1 NL4-3BaL were treated with DDX3i, SMACm, or their combination. This analysis was performed on a mixed population of infected and non-infected cells. In parallel, uninfected cells were treated with the same compounds. Six hours post-treatment, transcription of survival and apoptosis genes was analyzed by qPCR array. Of the 376 genes analyzed, 297 genes passed the data quality control analyses for detection of expression and off-target primer binding (Figure 3A). Hierarchical clustering of log2 fold gene expression relative to uninfected untreated cells was performed and included all genes that exhibited differential expression in at least one condition (log2 fold change greater than 1 or less than −1) (Figure 3B; Supplementary Tables S1–S7). HIV-1 infection alone reduced the expression of a large number of genes (Supplementary Table S4) and, interestingly, treatment of HIV-1 infected cells with DDX3i (Supplementary Table S7) restored the expression of many downregulated genes (Figure 3B). Uninfected cells treated with DDX3i formed a distinct cluster (Supplementary Table S1), separate from both HIV-1 infected cells with (Supplementary Table S7) or without DDX3i (Supplementary Table S4) and the SMACm conditions (Supplementary Tables S2, S3, S5 and S6). Uninfected cells treated with DDX3i exhibited the lowest number of differentially expressed genes compared to uninfected untreated cells (Figure 3B; Supplementary Table S1).

SMACm treatment had the strongest effect on gene expression, with all SMACm-treated conditions clustering together regardless of HIV-1 infection (Figure 3B; Supplementary Tables S2, S3, S5 and S6). Notably, the SMACm/DDX3i conditions were more closely related to SMACm-only than to DDX3i-only conditions. Overall, upregulation of the expression of genes involved in survival and apoptosis pathways was largely influenced by SMACm treatment, while HIV-1 infection induced downregulation of a subset of these genes. Interestingly, DDX3i demonstrated a more selective effect, specifically influencing genes downregulated by HIV-1.

### 3.4. DDX3i and SMACm Restore Expression of HIV-1 Downregulated Genes Involved in Apoptosis

**Figure 4:**
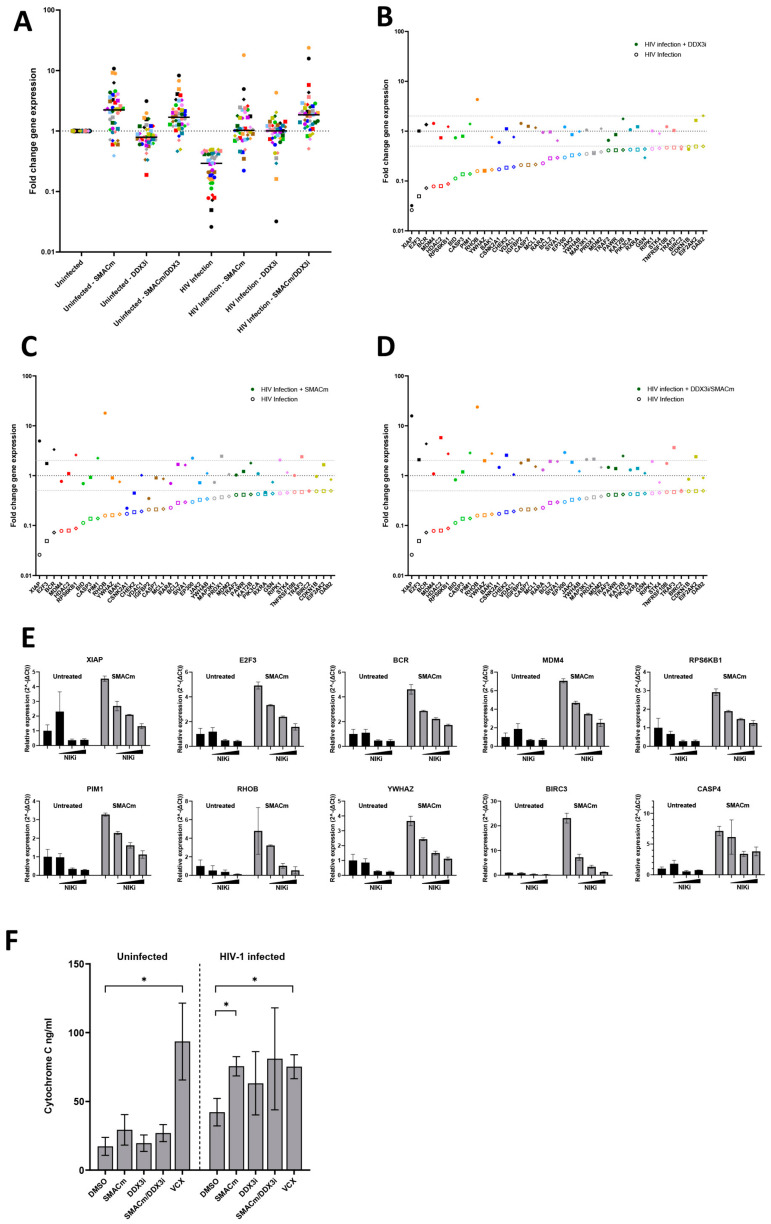
DDX3i and SMACm restore expression of genes involved in survival and apoptosis that are downregulated by HIV-1. Genes downregulated in SUPT1 by HIV-1 infection are selected for further analysis, and their expression in SUPT1 cells treated with or without SMACm, DDX3i, or the combination in the presence or absence of HIV-1 infection is shown (**A**). Expression levels of HIV-1 induced downregulated genes, ranked based on the level of downregulation, and the effect of DDX3i (**B**), SMACm (**C**) and DDX3i and SMACm combined (**D**) on the expression of these genes in HIV-1 infected SUPT1 cells is displayed. (**E**) Gene expression analysis by qPCR showing that the inhibition of NIK decreases SMACm-induced expression of XIAP, E2F3, BCR, MDM4, RPS6KB1, PIM1, RHOB, YWHAZ, BRC3 and CASP4 in SUPT1-CCR5 (N = 3). (**F**) Cytochrome C secretion of SUPT1-CCR5 cells (HIV-1 NL4-3BaL infected and uninfected) treated with SMACm (AZD5582; 1 µM), DDX3i (FH1321; 50 µM), a combination of both, or Venetoclax (10 µM) for 24 h was measured by ELISA (N = 3). Average and standard deviation of independent experiments are shown. Comparisons to untreated controls were made using a paired *t*-test, and significant events are displayed, * *p* < 0.05.

HIV-1 infection in SUPT1-CCR5 cells predominantly downregulated genes (41 genes down) (fold change < 0.5) involved in apoptosis and survival pathways (Figure 4A). The expression of the majority of these genes was restored by DDX3i and SMACm treatment, alone or in combination (Figure 4A). Interestingly, DDX3i increased gene expression of these genes only in the presence of HIV-1, whereas SMACm affected the expression of these genes irrespective of infection (Figure 4A). DDX3 inhibition restored the expression of most genes to baseline levels, and only the expression of RHOB was increased upon DDX3i treatment (Figure 4B). DDX3i did not restore HIV-1-mediated downregulation of XIAP, YWHAZ, PRDX1, GSN, cIAP1/BIRC2 and CDKN1B (Figure 4B). SMACm treatment, on the other hand, led to high expression of XIAP, BCR, RPS6KB1, PIM1, RHOB, EP300, PRDX1, RIPK1, and TRAF3 (Figure 4C). Moreover, the expression of five HIV-1 downregulated genes (CSNK2A1, CHEK2, IGFBP2, RXRA, cIAP1/BIRC2) was unaffected by SMACm treatment of HIV-1 infected cells. The combination treatment of DDX3i and SMACm led to upregulation of expression of all HIV-1 downregulated genes except for cIAP1/BIRC2. The expression of XIAP, E2F3, BCR, HDAC2, RPS6KB1, PIM1, RHOB, BAK1, CHEK2, EP300, KAT2B, TRAF3 and EIF2AK2 were highly expressed after DDX3i/SMACm treatment of HIV-1 infected cells (Figure 4D). These results indicate that both SMACm and DDX3i restore the expression of the majority of HIV-1 downregulated genes, and the combination of the compounds increases the expression of several HIV-1 downregulated genes above baseline levels. Reactome pathway analysis indeed confirmed that these genes were involved in pathways involving SMAC(DIABLO)-IAP, the intrinsic pathway for apoptosis, and the TNF receptor superfamily mediating non-canonical activation of the NF-kB pathway (Supplementary Figure S3).

As the expression of most of the genes downregulated by HIV-1 was restored by SMACm, it is likely that these genes are regulated through activation of the non-canonical NF-κB pathway and RelB. Therefore, we analyzed the effect of NIKi on genes that were downregulated by HIV-1 and induced by SMACm treatment (XIAP, E2F3, BCR, MDM4, RPS6KB1, PIM1, RHOB, YWHAZ). We also analyzed the effect of NIKi on the expression of cIAP2/BIRC3 and CASP4, whose expression increased upon SMACm treatment but was not affected by HIV-1. Notably, the SMACm-induced expression of all the genes was reversed by increasing concentrations of NIKi (Figure 4E), strongly indicating that SMACm regulates expression of these genes via RelB.

Next, we evaluated whether SMACm and DDX3i treatment made HIV-1-infected cells more sensitive to apoptosis. The release of cytochrome C from the mitochondria into the cytosol was measured as a surrogate for mitochondrial outer membrane permeabilization, as this triggers a signaling cascade that typically leads to apoptosis via the intrinsic apoptosis pathway, resulting in the activation of caspases 3 and 7 [30]. SUPT1-CCR5 cells, infected with HIV-1 NL4-3BaL or left uninfected for two days, were treated with DDX3i, SMACm, or their combination for 24 h before cells were lysed. HIV-1 infection alone induced cytosolic cytochrome C release compared to uninfected cells, which was further enhanced by DDX3i, SMACm, or the combination of both (Figure 4F). Venetoclax (VCX), a BCL-2 inhibitor, induced cytochrome C release irrespective of HIV-1 infection (Figure 4F). Our data show that DDX3i and SMACm likely enhance the sensitivity of HIV-1 infected cells to apoptosis.

## 4. Discussion

SMACms are promising agents for HIV-1 cure strategies and have been shown to reverse viral latency [26,27,31]. Interestingly, targeting IAPs with SMACm in combination with DDX3 inhibition reactivates latent HIV-1 and reduces the inducible latent reservoir in PBMCs from PWH ex vivo [13]. Here, we studied the underlying mechanism of targeting IAP and DDX3 on HIV-1 transcriptional activation combined with targeted qPCR array analysis for apoptotic and cell survival processes using an acute in vitro infection model.

In agreement with previous reports in cell lines as well as primary T cells [25,26,27], we observed that SMACm leads to activation of HIV-1 LTR-driven transcription both in the absence and presence of HIV-1 Tat. SMACm binds to cIAP1/BIRC2 and cIAP2/BIRC3, which play an important role in the regulation of the non-canonical NF-κB pathway [32]. Indeed, the SMACm AZD5582, as well as SBI-0637142, have been reported to activate the non-canonical NF-κB pathway by promoting the processing of p100 into p52 by NIK and p52 dimerization with RelB, leading to the formation of active RelB-p52 dimers [31]. We observed similarly that SMACm increased both canonical p65/p50 and RELB/p52 dimers. However, compared to TNF-α, a strong activator of both canonical and non-canonical NF-κB [33,34], SMACm was more efficient in activating the non-canonical NF-κB pathway. Although our data show that RelB/p52 is increased globally in the cells by SMACm and not specifically bound to the LTR, our data suggest that SMACm enhances HIV-1 transcription primarily through activation of RELB/p52 and to a lesser extent through p65/p50. Interestingly, our data suggest that SMACm initiates LTR-mediated HIV-1 transcription in the absence of HIV-1 Tat, which might be due to binding of RelB to the LTR, leading to transcription initiation and elongation. It is likely that RelB also activates other histone-modifying genes, such as histone acetyltransferases that might be involved in transcription elongation. It should be noted that we have used TZM-BL cells, which are not a latency model, as baseline LTR activity can be detected, and these cells are highly responsive to NF-κB activation and Tat [17]. Moreover, SMACm also enhances transcription in the presence of Tat, which might be explained by the activation of RelB and the subsequent recruitment of Tat and RNA Polymerase II to the promoter [25].

SMACm induced HIV-1 transcription in SUPT1-CCR5 cells infected with the dual reporter virus HIV-1 GKO/VSV-G. Although the transcriptionally inactive mKO2+GFP−negative cells are not equivalent to latent HIV-1 reservoir cells in vivo, our data suggest that the transcriptional activation of HIV-1 by SMACm might be involved in HIV-1 latency reversal, as we have observed previously in PBMCs from PWH ex vivo [13].

As NIK is a central regulator of the non-canonical activation of the NF-κB pathway [28,35], blocking of NIK abrogated SMACm-induced HIV-1 transcription, suggesting that RelB/p52 drives SMACm-induced HIV-1 transcription, even though we cannot exclude that NIK inhibition has an indirect effect on HIV-1 transcription. This confirms that SMACm acts as a potent LRA through activation of the non-canonical NF-κB pathway [26,27,31]. Importantly, our data show that RelB activation by SMACm is not only involved in HIV-1 transcription but also upregulates anti- and pro-apoptotic genes that are suppressed during HIV-1 infection (Supplementary Figure S4). Moreover, we observed that inhibition of DDX3 using FH1321 neither induced viral transcription nor enhanced SMACm-induced HIV-1 transcription, confirming our previous observations [13]. These data are in contrast to those of Rao et al. [11], who show that DDX3i induces HIV-1 transcription in latently infected cells. These differences might be due to the use of different inhibitors and/or cell models.

Many LRA classes, like protein kinase C (PKC) agonists, mitogen-activated protein kinase (MAPK) agonists, protein kinase B (Akt) pathway activators and Toll-like receptor agonists, induce HIV-1 latency reversal directly or indirectly through the activation of the canonical NF-κB pathway [36]. However, this pathway is a major regulator of genes involved in inflammatory and innate responses [37,38]. Therefore, LRA activation of canonical NF-κB may affect or accelerate HIV-1 associated inflammation and consequently, contribute to immune dysfunction and comorbidity development in PWH [39]. Activation of the non-canonical NF-κB pathway leads to the regulation of a more limited set of genes related to the adaptive immune response [38], and therefore, activation of this pathway for latency reversal might be more favorable. Indeed, non-canonical activation of the NF-κB pathway using SMACm did not result in the induction of systemic inflammation in resus macaques [31,40]. In SUPT1, we observed that the SMACm AZD5582 strongly increased the expression of several genes like cIAP2/BIRC3, IL-10, IFNγ and CASP4 irrespective of DDX3i treatment and HIV-1 infection (Supplementary Tables S2, S3, S5 and S6), implicating controlled immune activation. The increase in IFNγ and NF-κB-driven CASP4 expression is reflective of immune activation [41,42,43], while the increased NF-κB-regulated expression of cIAP2/BIRC3 and IL-10 suggests re-establishment of apoptotic restraint [29,44] and suppression of inflammation [45].

SMACm has been shown to restore the sensitivity of cancer cells to apoptotic stimuli. SMACm binds to BIR3 domains of IAPs, including XIAP/BIRC4, cIAP1/BIRC2 and cIAP2/BIRC3, and inhibits their anti-apoptotic function [29,44]. XIAP prevents apoptosis through direct interaction and inhibition of caspase 3, 7 and 9 [46,47]. cIAP1/BIRC2 and cIAP2/BIRC3 also prevent apoptosis by a different mechanism involving E3 ubiquitin ligase-mediated TRAF2 degradation and canonical NF-κB-regulated expression of genes involved in cell survival [48]. Using a PCR-array for genes involved in apoptosis and cell survival, we observed indeed that SMACm treatment of SUPT1 cells resulted in a strong induction of the expression of apoptosis-related genes, irrespective of HIV-1 infection, suggesting that SMACm in the absence of HIV-1 can sensitize genes to apoptosis. However, our data strongly suggest that SMACm specifically induces apoptosis in HIV-1 infected cells [13].

HIV-1 is known to modulate apoptotic pathways to promote viral replication and dissemination. We observed that HIV-1 infection suppressed expression of several pro-apoptotic genes (such as BID, CASP3, RHOB, BAK1, CASP7, VDAC1 and SIVA1), as well as anti-apoptotic genes (such as XIAP, BCL-2, MCL1 and PIM1) and genes promoting cell survival by p53 suppression (MDM2, MDM4, HDAC2, YWHAZ and YWHAB). Suppression of these pro- and anti-apoptotic pathways by HIV-1 viral proteins has been described previously and is likely dependent on the differential expression of these proteins during distinct phases of viral production [49,50]. Early after initiation of viral transcription, low-level HIV-1 protein production from mainly (multiple) spliced mRNAs is observed. Low levels of HIV-1 Tat, Nef, and Vpr produced during this phase have been associated with anti-apoptotic functions and support prolonged survival of the infected cell [51,52,53,54]. Pro-apoptotic effects have been described for HIV-1 Env, protease, Vpu and high levels of Tat, Nef and Vpr to support viral release and immune evasion of infected cells [51,55,56,57,58,59,60]. In our assay, we have used SUPT1 cells acutely infected with replicating HIV-1. These cultures likely contain a mixture of infected cells during all phases of infection and viral production, as well as uninfected bystander cells in which gene expression is affected by exposure to viral proteins. This likely explains why we observed dysregulation of pro-apoptotic, anti-apoptotic, and cell survival genes.

Interestingly, HIV-1 downregulated expression of genes involved in apoptosis and cell survival was almost completely restored by SMACm treatment. This suggests that IAPs and non-canonical activation of NF-κB may be strong regulators of the HIV-1 regulated gene expression. Notably, we observed that the inhibition of NIK abrogated SMACm-induced expression of these genes. These data thereby strongly suggest that non-canonical activation of the NF-κB pathway not only activates HIV-1 transcription but can also regulate apoptosis sensitivity of HIV-1 infected cells.

Although DDX3 inhibition in SUPT1 cells only had a minor effect on the expression of genes involved in apoptosis and cell survival, notably, DDX3i specifically restored expression of most genes downregulated by HIV-1, except for XIAP, YWHAZ, PRDX1, GSN, cIAP1/BIRC2 and CDKN1B. The combination of SMACm and DDX3i restored gene expression of almost all HIV-1 downregulated genes, with only cIAP1/BIRC2 levels remaining suppressed. These data suggest that the combination of SMACm and DDX3i especially increases apoptosis sensitivity of HIV-1 infected cells through normalizing the expression of HIV-1 downregulated genes involved in apoptosis and cell survival. We have validated important genes targeted by SMACm-induced RelB by qPCR. We did not specifically validate the expression of the proteins involved in apoptosis and cell survival, and the net result of the observed regulation of pro- and anti-apoptotic genes on cell death remains unclear. However, we observed that SMACm alone or in combination with DDX3i increased cell death of HIV-1 infected cells in cytosolic cytochrome C release, and together with our previous data [13], this suggests that the net effect will lead to cell death of HIV-1 infected cells. These data indicate that these treatments increase the susceptibility of HIV-1 infected cells to apoptosis through restored expression of apoptosis-related genes and cytosolic cytochrome C, which also sensitizes cells further to cell death.

Latently infected cells only support low-level HIV-1 transcription and protein production, and therefore their gene expression profile is likely associated with an anti-apoptotic state and increased resistance to apoptosis [61,62,63]. SMACm and DDX3i are able to disrupt the HIV-1 induced balance in the expression of genes involved in apoptosis and cell survival and can specifically induce death of the HIV-1 reservoir ex vivo [13]. Notably, the use of DDX3i, which demonstrated specificity of gene regulation in HIV-1 infected cells in an acute in vitro infection model, may account for the specific death of HIV-1 infected cells observed in primary cell cultures treated by the combination of DDX3i and SMACm using ex vivo PBMC from PWH [13].

We have chosen an acute in vitro infection cell-line model to investigate the mechanisms behind DDX3i and SMACm in HIV-1 transcription and apoptosis sensitivity of HIV-1 infected cells. Immortalized cells are less sensitive to HIV-1 induced apoptosis due to elevated levels of anti-apoptotic molecules, including BCL2, BCL-XL and MCL1 [64,65], and functional impairment of the p53 pathway [66]. This provided a time window for analyzing anti- and pro-apoptotic gene expression profiles that would have been more complicated in primary T cells. Moreover, SUPT1-CCR5 cells originate from a T lymphoblastic leukemia and exhibit an immature phenotype with high proliferative ability [67], whereas primary CD4 T cells are highly heterogeneous. CD4 cells are long-lived, and homeostasis is controlled by a balance between proliferation and apoptosis. Cytokines, including IL-2, IL-7 and IL-15, support self-renewal of memory and stem-like memory populations, while antigen triggering of the T cell receptors induces clonal expansion and effector differentiation [68]. These differences regarding the differentiation and proliferative state will likely alter apoptotic pathways and the net balance of pro- and anti-apoptotic gene expression. However, our findings corroborate our previous data, showing that DDX3i and SMACm induce specific apoptosis of HIV-1 infected cells in PBMC from PWH without inducing cell death in uninfected T cells and non-T cell types [13].

We have chosen an acute infection model with replication-competent HIV-1 rather than latency models such as J-Lat cell lines. J-Lat cells are clonal cell lines, each harboring a single, integrated, replication-incompetent HIV-1 provirus at a fixed genomic site. As a result, these latency models do not capture HIV-1 reactivation across diverse integration environments and generate replication-incompetent viruses after reactivation. Moreover, the lack of HIV-1 genes such as Env and Nef might not recapitulate reactivation of replicating HIV-1 and subsequent effects on gene expression, including those involved in apoptosis and cell survival. Further studies are warranted to determine what pro- and anti-apoptotic genes are regulated by SMACm and DDX3i in HIV-1 latent infected cells.

Here, we show that SMACm induces HIV-1 transcription through the non-canonical activation of the NF-κB pathway. Additionally, we show that both SMACm and DDX3i restore the expression of apoptosis-related genes that are specifically suppressed by HIV-1, altering the apoptotic susceptibility of HIV-1 infected cells. Our findings suggest that a combination of compounds that can activate HIV-1 transcription and reverse HIV-1 induced anti-apoptotic mechanisms may promote targeted cell death of HIV-1 infected cells and contribute to the advancement of HIV-1 cure strategies.

## Data Availability

The datasets generated and analyzed during the current study are available in the Gene Expression Omnibus repository (https://www.ncbi.nlm.nih.gov/geo/, 15 February 2026), accession number GSE303821.

## Supplementary Materials

The following supporting information can be downloaded at: https://www.mdpi.com/article/10.3390/pathogens15060575/s1, Figure S1: SMACm activates the HIV-1 LTR in the absence of Tat; Figure S2: Reactome pathway analysis; Figure S3: Schematic function of SMACm and DDX3i on HIV-1 reactivation and induction of apoptosis; Figure S4: Schematic function of SMACm and DDX3i on HIV-1 reactivation and induction of apoptosis; Table S1: Differentially expressed genes in uninfected SUPT1-CCR5 cells treated with DDX3i; Table S2: Differentially expressed genes in uninfected SUPT1-CCR5 cells treated with SMACm; Table S3: Differentially expressed genes in uninfected SUPT1-CCR5 cells treated with DDX3i and SMACm; Table S4: Differentially expressed genes in SUPT1-CCR5 cells infected with HIV-1; Table S5: Differentially expressed genes in HIV-1–infected SUPT1-CCR5 cells treated with SMACm; Table S6: Differentially expressed genes in HIV-1–infected SUPT1-CCR5 cells treated with DDX3i and SMACm; Table S7: Differentially expressed genes in HIV-1–infected SUPT1-CCR5 cells treated with DDX3i; Table S8: Primers used for qPCR.

## Abbreviations

The following abbreviations are used in this manuscript:

HIV-1	Human Immunodeficiency Virus 1
SMACm	Second mitochondrial-derived activator of caspases mimetics
LRA	Latency reversal agent
PWH	People living with HIV-1
ART	Antiretroviral therapy
NF-κB	Nuclear factor kappa-light-chain-enhancer of activated B cells
VSV-G	Vesicular Stomatitis Virus Glycoprotein
EF1α	Elongation Factor 1 Alpha
mKO2	Monomeric Kusabira-Orange 2
NIK	NF-κB–Inducing Kinase
LTR	Long terminal repeat
FCS	Fetal calf serum
PBMC	Peripheral Blood Mononuclear Cells
DDX3	DEAD-Box Helicase 3
DMSO	Dimethylsulfoxide
GFP	Green fluorescent protein
TNF-α	Tumor necrosis factor
IMDM	Iscove’s Modified Dulbecco’s Medium
XIAP	X-linked Inhibitor of Apoptosis Protein
E2F3	E2F Transcription Factor 3
BCR	Breakpoint cluster region
MDM4	Mdm4 p53 binding protein homolog
RPS6KB1	Ribosomal Protein S6 Kinase B1
PIM1	Pim-1 Proto-Oncogene
RHOB	Ras Homolog Family Member B
YWHAZ	Tyrosine 3-Monooxygenase/Tryptophan 5-Monooxygenase Activation Protein Zeta
BIRC3	Baculoviral IAP repeat containing 3
CASP4	Caspase 4
BID	BH3 Interacting Domain Death Agonist
CDKN1B	Cyclin-Dependent Kinase Inhibitor 1B
CASP3	Caspase 3
BAK1	BCL-2 Antagonist/Killer 1
BCL-2	B-Cell Lymphoma 2
PRDX1	Peroxiredoxin 1
GSN	Gelsolin
YWHAB	Tyrosine 3-Monooxygenase/Tryptophan 5-Monooxygenase Activation Protein Beta
HDAC2	Histone Deacetylase 2
MDM2	Mdm2 p53 binding protein homolog
MCL-1	Myeloid Cell Leukemia 1
SIVA1	SIVA Apoptosis Inducing Factor 1
BIRC2	Baculoviral IAP Repeat Containing 2
VDAC1	Voltage-dependent anion channel 1
CASP7	Caspase 7
IFNγ	Interferon Gamma
cIAP	Cellular Inhibitor of Apoptosis Protein
VCX	Venetoclax
