# Supplementary material for: Blocking Host Factors IAP and DDX3 Activates HIV-1 Transcription and Increases Apoptosis Sensitivity of HIV-1 Infected Cells
Source: Pathogens. 2026 May 27;15(6):575. doi: 10.3390/pathogens15060575 (PMC13306193; doi:10.3390/pathogens15060575)
Supplement: Supplementary file 1 [file pathogens-15-00575-s001.zip › pathogens-4185289-supplementary.pdf]

Supplementary information file

Blocking Host Factors IAP and DDX3 Reverse HIV-1 Latency and Restore Apoptosis Vulnerability of the HIV-1 reservoir

Jade Jansen[1,2], Shirley Man[1,2], Fenna Kootstra[1,2], Ad C. van Nuenen[1,2], Karel A. van Dort[1,2], Claudio Zamperini[3], Conraed Willem Houck[3], Neeltje A. Kootstra[1,2]#, Teunis B. H. Geijtenbeek[1,2]#

Supplementary figures

Figure S1. SMACm activates the HIV-1 LTR in the absence of Tat.

Figure S2. Caspase3/7 activity following treatment with SMACm and/or DDX3i.

Figure S3. Reactome pathway analysis

Figure S4. Schematic function of SMACm and DDX3i on HIV-1 reactivation and induction of apoptosis.

Supplementary tables

Table S1. Differentially expressed genes in uninfected SUPT1-CCR5 cells treated with DDX3i.

Table S2. Differentially expressed genes in uninfected SUPT1-CCR5 cells treated with SMACm.

Table S3. Differentially expressed genes in uninfected SUPT1-CCR5 cells treated with DDX3i and SMACm.

Table S4. Differentially expressed genes in SUPT1-CCR5 cells infected with HIV-1.

Table S5. Differentially expressed genes in HIV-1-infected SUPT1-CCR5 cells treated with SMACm.

Table S6. Differentially expressed genes in HIV-1-infected SUPT1-CCR5 cells treated with DDX3i and SMACm.

Table S7. Differentially expressed genes in HIV-1-infected SUPT1-CCR5 cells treated with DDX3i.

Table S8. Primers used for qPCR.

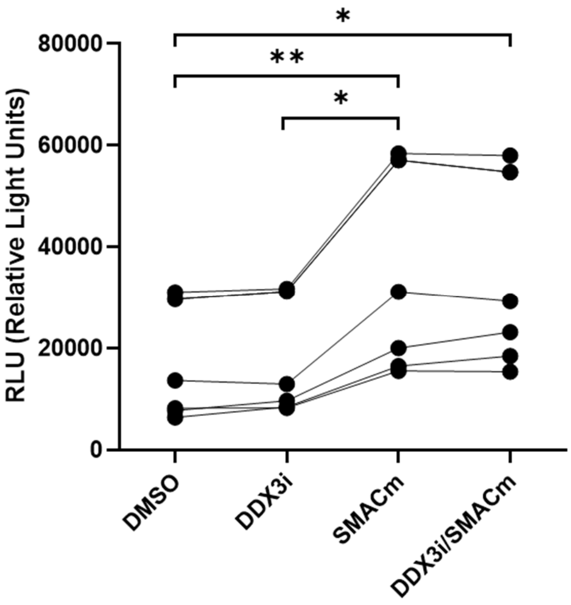

Figure S1. SMACm activates the HIV-1 LTR in the absence of Tat.

TZM-BL cells were cultured with SMACm AZD5582 (1µM), DDX3i FH1321 (50µM), both or DMSO for 48 hours and HIV-1 LTR activation was assessed by luminescence, reported in relative light units (RLU). Comparisons to each condition were made using ANOVA. 6 independent experiments are shown, *$p < 0.05$, **$p < 0.01$.

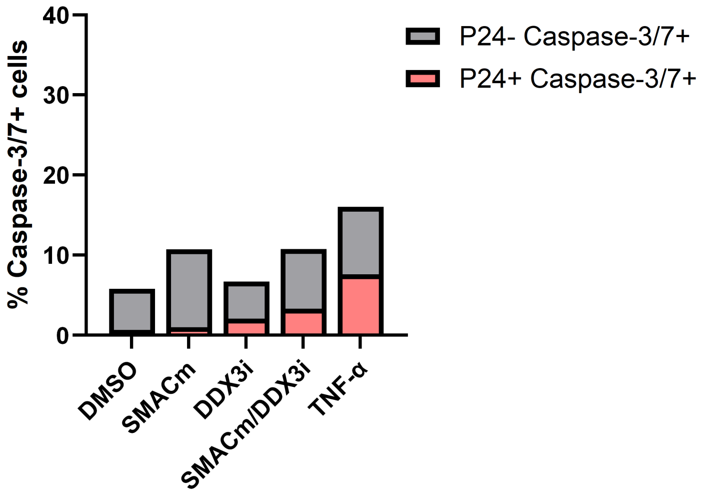

Figure S2. Caspase3/7 activity following treatment with SMACm and/or DDX3i.

SUPT1-CCR5 cells were infected with HIV-1 NL4-3BaL for 36 hours and subsequently treated with SMACm (AZD5582; 1 µM), DDX3i (FH1321; 50 µM) or both. Caspase-3/7 activity was measured by flow cytometry at 24 hr post-treatment in p24- and p24+ cell populations (N=1).

Pathway	Genes found	Genes total	Interactions found
SMAC (DIABLO)-mediated dissociation of IAP-caspase complexes	4	7	0
Intrinsic Pathway for Apoptosis	10	64	19
TNFR1-induced pro-apoptotic signaling	5	26	4
CASP8 activity is inhibited	3	12	0
Activation of BAD and translocation to mitochondria	4	19	6
TNF receptor superfamily (TNFSF) members mediating non-canonical NF-kB pathway	4	17	3
Erythropoietin activates Phosphoinositide-3-kinase (PI3K)	3	16	1
SMAC (DIABLO) binds to IAPs	4	7	5
SMAC, XIAP-regulated apoptotic response	4	8	5

Figure S3. Reactome pathway analysis.

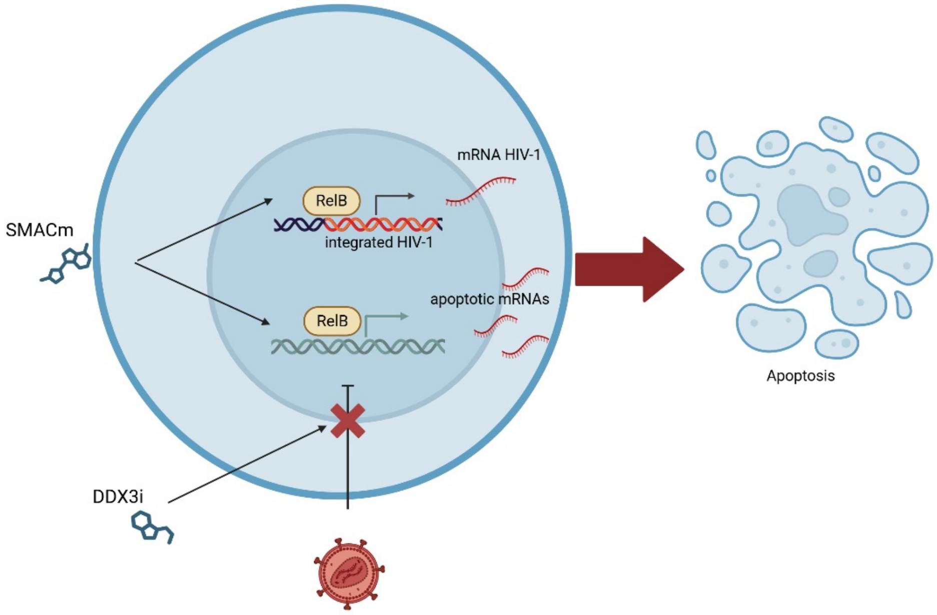

Figure S4. Schematic function of SMACm and DDX3i on HIV-1 reactivation and induction of apoptosis.

Table S1. Differentially expressed genes in uninfected SUPT1-CCR5 cells treated with DDX3i. Listed are all differentially expressed genes, with a fold change <0.5 or >2, in DDX3i-treated cells. Fold changes were calculated relative to the uninfected and DMSO-treated control.

Fold change	Gene
0.158586	*CYBA*
0.188591	*MDM4*
0.335256	*MCL1*
0.398688	*DHCR24*
0.427304	*BIRC2*
0.464366	*TNF*
0.470848	*GADD45G*
0.477421	*PRKCH*
0.477421	*SFN*
0.497695	*BTK*
0.497695	*MDM2*
2.19365	*CYCS*
2.417194	*F3*
2.555018	*EPO*
3.123868	*XIAP*

Table S2. Differentially expressed genes in uninfected SUPT1-CCR5 cells treated with SMACm. Listed are all differentially expressed genes, with a fold change <0.5 or >2, in SMACm-treated cells. Fold changes were calculated relative to the uninfected and DMSO-treated control.

Fold change	Gene
0.233258	*UBB*
0.389582	*YWHAB*
0.395021	*TOP2A*
0.432269	*EPHA2*
0.486327	*PRKCZ*
0.493116	*MAP2K6*
2	*HTATIP2*
2	*MYC*
2	*HRAS*
2	*PPARD*
2.013911	*BCL10*
2.027919	*ERBB3*
2.027919	*CASP9*
2.042024	*EIF2AK3*
2.056228	*ENDOG*
2.056228	*APC*
2.056228	*HSPA5*
2.056228	*IKBKE*
2.056228	*JAK2*
2.07053	*BAD*
2.084932	*PRKDC*
2.084932	*ATF2*
2.099433	*KAT2B*
2.114036	*BTRC*
2.114036	*IFI27*
2.143547	*KAT5*
2.143547	*PML*
2.143547	*JUNB*
2.17347	*ERN1*
2.20381	*MAPK7*
2.219139	*KEAP1*
2.219139	*PHLPP1*
2.219139	*RPS6KB1*
2.234574	*STK4*
2.265768	*ABL1*
2.265768	*CASP10*
2.281527	*CDKN1B*
2.281527	*NFKB1*
2.297397	*HTRA2*
2.297397	*EIF2AK2*
2.297397	*JUN*
2.329467	*PRODH*
2.34567	*BID*
2.361985	*SIVA1*
2.378414	*ATF4*
2.378414	*RAC1*
2.411616	*PTK2B*
2.42839	*CSNK2A1*

2.42839	*TRAF5*
2.445281	*EGLN3*
2.496661	*NCF1*
2.496661	*MAP3K7*
2.496661	*TP53*
2.584706	*TRAF3*
2.732081	*PEA15*
2.770219	*SMAD3*
2.789487	*CASP6*
2.80889	*STK11*
2.80889	*TNFRSF1B*
2.8481	*ITGB2*
2.86791	*PRKCD*
2.887858	*CYCS*
2.907945	*TNFAIP3*
2.928171	*FOXO1*
2.948538	*HDAC2*
3.052518	*MAX*
3.052518	*THBS1*
3.09513	*IGFBP2*
3.160165	*TNFRSF1A*
3.160165	*E2F1*
3.182146	*BCL2*
3.226567	*CLU*
3.271608	*E2F3*
3.317278	*SRC*
3.41054	*DAXX*
3.434262	*MALT1*
3.555371	*PIK3CD*
3.5801	*TRAF6*
3.605002	*EP300*
3.680751	*FGFR3*
3.758091	*BAK1*
3.810552	*MAPK11*
3.97237	*CHEK2*
3.97237	*EPO*
4.084049	*RIPK1*
4.346939	*TRAF2*
4.531536	*MAPK3*
4.563055	*PIM1*
4.594793	*CD44*
4.958831	*TRAF1*
5.098243	*F3*
6.276673	*BCR*
7.568461	*STAT1*
8.938297	*RHOB*
9.189587	*YWHAZ*
10.77787	*XIAP*
22.7848	*IL10*
24.76104	*CASP4*
28.84001	*BIRC3*
119.4282	*IFNG*

Table S3. Differentially expressed genes in uninfected SUPT1-CCR5 cells treated with DDX3i and SMACm. Listed are all differentially expressed genes, with a fold change <0.5 or >2, in cells treated with DDX3i and SMACm. Fold changes were calculated relative to the uninfected and DMSO-treated control.

Fold change	Gene
0.255253	*IGFBP5*
0.257028	*EGF*
0.351111	*EPHA2*
0.493116	*ID1*
0.496546	*DAB2*
2	*BCL2*
2.013911	*MAX*
2.042024	*TRAF3*
2.12874	*MALT1*
2.143547	*CHEK2*
2.17347	*CYCS*
2.219139	*EIF2AK2*
2.250117	*PRODH*
2.281527	*TRAF6*
2.313376	*PIK3CD*
2.313376	*BAK1*
2.329467	*RPS6KB1*
2.34567	*DAXX*
2.584706	*BAG3*
2.639016	*MAPK9*
2.675855	*RIPK1*
2.675855	*PIM1*
2.751084	*MAPK3*
2.80889	*ATM*
2.969047	*CD44*
2.989698	*THBS1*
3.182146	*EP300*
3.24901	*SIVA1*
3.24901	*TRAF1*
3.317278	*CDH1*
3.317278	*BCR*
3.89062	*HDAC2*
4.084049	*E2F3*
4.084049	*F3*
4.924578	*YWHAZ*
5.979397	*STAT1*
6.773962	*RHOB*
8.282119	*XIAP*
14.22148	*BIRC3*
15.56248	*CASP4*
29.65082	*GZMB*
80.44886	*IFNG*

Table S4. Differentially expressed genes in SUPT1-CCR5 cells infected with HIV-1. Listed are all differentially expressed genes, with a fold change <0.5 or >2, in HIV-1-infected and DMSO-treated cells. Fold changes were calculated relative to the uninfected and DMSO-treated control.

Fold change	Gene
0.026036	*XIAP*
0.049264	*E2F3*
0.072126	*BCR*
0.077841	*MDM4*
0.078927	*HDAC2*
0.087575	*RPS6KB1*
0.112396	*BID*
0.136471	*CASP3*
0.138376	*PIM1*
0.157854	*RHOB*
0.160058	*YWHAZ*
0.168016	*BAK1*
0.171546	*CSNK2A1*
0.185137	*CHEK2*
0.191666	*VDAC1*
0.20829	*IGFBP2*
0.209739	*CASP7*
0.214146	*MCL1*
0.226356	*RARA*
0.284533	*BCL2*
0.292532	*SIVA1*
0.296616	*EP300*
0.326842	*JAK2*
0.340722	*YWHAB*
0.350301	*MAP3K1*
0.367717	*PRDX1*
0.383332	*MDM2*
0.410845	*TRAF2*
0.413703	*PAWR*
0.419478	*KAT2B*
0.425334	*PIK3CA*
0.425334	*RXRA*
0.437291	*GSN*
0.440333	*RIPK1*
0.452712	*STK4*
0.46544	*TNFRSF10B*
0.468677	*TRAF3*
0.481854	*BIRC2*
0.485205	*CDKN1B*
0.491978	*EIF2AK2*
0.4954	*DAB2*
2.168454	*MAPK11*
2.292095	*PIK3CD*
2.308038	*CYCS*
2.308038	*RAC1*
2.706947	*NFKB1*
3.547166	*CDH1*
3.571838	*THBS1*

4.745851	*F3*

Table S5. Differentially expressed genes in HIV-1-infected SUPT1-CCR5 cells treated with SMACm. Listed are all differentially expressed genes, with a fold change <0.5 or >2, in HIV-1-infected and SMACm-treated cells. Fold changes were calculated relative to the uninfected and DMSO-treated control.

Fold change	Gene
0.221698	*CSNK2A1*
0.347881	*IGFBP2*
0.41658	*TP53*
0.431271	*MAP2K6*
0.431271	*PIK3CG*
0.443396	*TGFBR2*
0.44648	*CHEK2*
0.459032	*RXRA*
0.491978	*BIRC2*
0.498846	*TMBIM6*
2.009263	*BAG3*
2.009263	*MAPK9*
2.037312	*ERBB3*
2.051482	*RIPK1*
2.1386	*IFNB1*
2.168454	*SMAD3*
2.244924	*PIK3CD*
2.244924	*EP300*
2.244924	*PIM1*
2.276262	*MAPK3*
2.340257	*TRAF5*
2.38943	*SRC*
2.38943	*TRAF3*
2.422785	*NFKB1*
2.456606	*PRDX1*
2.473693	*CLU*
2.560928	*CYCS*
2.596677	*RPS6KB1*
2.614738	*ATM*
2.706947	*FOXO1*
2.744735	*MAPK11*
3.087987	*TNFRSF1B*
3.332643	*BCR*
3.402669	*TRAF1*
3.450168	*CD44*
3.935827	*F3*
4.428035	*THBS1*
4.947387	*XIAP*
8.205929	*STAT1*
12.87648	*CASP4*
17.58979	*IL10*
17.95939	*RHOB*
24.87572	*BIRC3*
75.93361	*IFNG*

Table S6. Differentially expressed genes in HIV-1-infected SUPT1-CCR5 cells treated with DDX3i and SMACm. Listed are all differentially expressed genes, with a fold change <0.5 or >2, in HIV-1-infected and combination-treated cells. Fold changes were calculated relative to the uninfected and DMSO-treated control.

Fold change	Gene
0.31864	*EGF*
0.371131	*MAP2K6*
0.384219	*EPHA2*
0.426317	*TGFBR2*
0.447513	*PYCARD*
0.5	*ABL1*
2.013911	*PPP2R5C*
2.056228	*MAX*
2.056228	*PRKCE*
2.056228	*CASP7*
2.084932	*TRAF5*
2.084932	*E2F3*
2.099433	*MAP3K1*
2.114036	*MAPK11*
2.114036	*APAF1*
2.143547	*PRDX1*
2.188587	*HTATIP2*
2.234574	*DAPK1*
2.250117	*JUN*
2.281527	*MAPK3*
2.34567	*NCF1*
2.361985	*PIK3CD*
2.378414	*SRC*
2.394957	*EIF2AK2*
2.42839	*PEA15*
2.445281	*CYCS*
2.479415	*KAT2B*
2.479415	*NFKB1*
2.479415	*FGFR3*
2.514027	*ERBB3*
2.549121	*PRKCD*
2.566852	*CHEK2*
2.620787	*TNFRSF1B*
2.639016	*TRAF6*
2.732081	*RPS6KB1*
2.770219	*BAK1*
2.8481	*PIM1*
2.907945	*EP300*
2.948538	*MAPK9*
2.969047	*CLU*
3.010493	*MALT1*
3.138336	*THBS1*
3.340352	*FOXO1*
3.340352	*TRAF1*
3.363586	*ATM*
3.530812	*BAG3*

3.655326	*TRAF3*
4.14106	*EPO*
4.287094	*CD44*
4.346939	*BCR*
5.775717	*HDAC2*
6.19026	*F3*
8.282119	*STAT1*
8.876556	*CDH1*
14.723	*IL10*
15.77972	*XIAP*
22.47112	*BIRC3*
23.75238	*RHOB*
26.72281	*CASP4*
79.34129	*IFNG*

Table S7. Differentially expressed genes in HIV-1-infected SUPT1-CCR5 cells treated with DDX3i. Listed are all differentially expressed genes, with a fold change <0.5 or >2, in HIV-1-infected and DDX3i-treated cells. Fold changes were calculated relative to the uninfected and DMSO-treated control.

Fold change	Gene
0.032054	*XIAP*
0.084592	*TNFRSF25*
0.161171	*YWHAZ*
0.355191	*PRDX1*
0.362654	*DAXX*
0.431271	*CDKN1B*
0.434271	*BIRC2*
2.037312	*DAB2*
2.051482	*MAPK9*
2.08012	*MAX*
2.1386	*ITGB2*
2.198724	*NFKB1*
2.198724	*PIK3CD*
2.198724	*DAPK1*
2.543238	*RAC1*
2.962195	*CYCS*
3.828201	*CDH1*
4.30695	*RHOB*
4.367073	*F3*
5.051342	*THBS1*

Table S8. Primers used for qPCR

Gene		Sequence (5' → 3')
XIAP	Forward	AATAGTGCCACGCAGTCTACA
	Reverse	CAGATGGCCTGTCTAAGGCAA
E2F3	Forward	GTATGATACGTCTCTTGGTCTGC
	Reverse	CAAATCCAATACCCCATCGGG
BCR	Forward	TACCAGAGCATCTACGTCGGG
	Reverse	CCTCCGCAATCCTCAAAACTC
MDM4	Forward	TGATTGTCGAAGAACCATTTCGG
	Reverse	TGCAGGGATCAAAAAGTTTGGAG
RPS6KB1	Forward	AGAACTTCTGGCTCGAAAGGT
	Reverse	CGACAGGTGTCTGACGTGTAA
PIM1	Forward	GAGAAGGACCGGATTTCCGAC
	Reverse	CAGTCCAGGAGCCTAATGACG
RHOB	Forward	GCCATAAGCGAACTTTGTGC
	Reverse	CGAGTGCATTAAGTGTCAAACA
YWHAZ	Forward	CCTGCATGAAGTCTGTAACTGAG
	Reverse	GACCTACGGGCTCCTACAACA
BIRC3	Forward	TTTCCGTGGCTCTTATTCAAACT
	Reverse	GCACAGTGGTAGGAACTTCTCAT
CASP4	Forward	TCCGAATATGGAGGCTGGAC
	Reverse	CGTGTGCGGTTGTTTCTC
GAPDH	Forward	CGAGCCACATCGCTCAGACACC
	Reverse	CAAATGAGCCCCAGCCTTCTCCATG
β-actin	Forward	GGGTCAGAAGGATTCCTATG
	Reverse	GGTCTCAAACATGATCTGGG
Gag (US)	Forward	TGGGAAAAAATTCGGTTAAGGCC
	Reverse	CTTCTACTACTTTTACCCATGC
Tat/Rev (MS)	Forward	CTTAGGCATCTCCTATGGCAGGAA
	Reverse	TTCCTTCGGGCCTGTCGGGTCCC
Env (SS)	Forward	GGAGCAGCAGGAAGCACTAT
	Reverse	AGATGCTGTTGCGCCTCAAT
